# The ‘triradiate bump’: a novel radiographic sign that may confound assessment of acetabular retroversion

**DOI:** 10.1007/s11832-016-0737-5

**Published:** 2016-04-28

**Authors:** William Z. Morris, Ryan T. Li, Raymond W. Liu

**Affiliations:** Division of Pediatric Orthopaedics, Rainbow Babies and Children’s Hospitals, Case Western Reserve University, 11100 Euclid Avenue, RBC 6081, Cleveland, OH 44106 USA; Department of Orthopaedic Surgery, Case Western Reserve University, 11100 Euclid Avenue, HH 504, Cleveland, OH 44106 USA

**Keywords:** Acetabular retroversion, Ischial spine sign, Hip development, Radiographic study, Femoroacetabular impingement

## Abstract

**Purpose:**

The triradiate cartilage transiently projects medially within the pelvic brim around the time of triradiate closure, mimicking the ischial spine sign. The purpose of this study was to characterize this newly identified radiographic sign using a longitudinal radiographic study.

**Methods:**

We identified 72 subjects from a longitudinal radiographic study of healthy adolescents, each with at least four consecutive, annual anterior−posterior radiographs of the left hip, including physeal closure. Images were reviewed to identify the presence of the triradiate bump, the year it was most prominent, and the number of years relative to triradiate closure after which it had completely remodeled.

**Results:**

The transient medial projection of the triradiate cartilage (triradiate bump) was identified in 26/40 (65 %) females and 22/32 (69 %) males (*p* = 0.74). The medial projection of the triradiate cartilage was most prominent at 10.8 ± 0.8 years of age in females and 12.6 ± 0.7 years of age in males (*p* < 0.001). The triradiate cartilage projected medially a mean of 4.7 ± 0.8 or 5.1 ± 1.4 mm for females and males, respectively (*p* = 0.29), but remodeled completely in all cases around triradiate closure.

**Conclusions:**

The transient medial projection of the triradiate cartilage within the pelvic brim, the ‘triradiate bump sign’, is a common radiographic finding in healthy adolescents around the time of closure of the triradiate cartilage that may mimic the ischial spine sign. These two signs can be distinguished as the projection of the ischial spine is located more inferiorly within the pelvic brim and the triradiate bump has a horizontal limb of radiolucency extending to its medial border.

## Introduction

Femoroacetabular impingement (FAI) is a frequent source of hip pain in adolescents and young adults. Increasing attention has been focused on the evaluation of adolescents over the past decade as studies have revealed that adolescents involved in vigorous sporting activity are at an increased risk for the development of cam morphology in FAI [1–6]. While isolated cam (or pincer) may cause FAI, many patients instead present with a mixed ‘cam and pincer’ deformity, where hip morphology is notable for both decreased femoral head/neck offset and acetabular retroversion. Acetabular retroversion increases the likelihood of mechanical impingement with hip flexion and internal rotation [7, 8] which can subsequently lead to damage to the anterior labrum and adjacent cartilage [9]. Consequently, evaluation of an adolescent patient with hip pain involves thorough clinical and radiographic assessment for acetabular retroversion.

Radiographic assessment of acetabular retroversion includes evaluation for the crossover sign (COS) and the ischial spine sign (ISS). These two radiographic markers are present on anterior−posterior (AP) pelvis plain films and are reflective of cranial acetabular retroversion. Jamali et al. [10] first described the COS as a marker of acetabular retroversion seen when the anterior wall of the acetabulum extends further laterally than the posterior wall on an AP pelvis radiograph. Kalberer et al. [11] first identified the ISS as the medial projection of the ischial spine within the pelvic brim. They demonstrated that the ISS was strongly predictive of acetabular retroversion (sensitivity 91 %, specificity 98 %) and proposed that it is simpler to evaluate than the COS as it relies only on identifying “a triangular-shaped radio-opaque structure that points medially from the pelvic brim toward the pelvic inlet” [11]. While elegant in its simplicity, the ISS may be prone to misinterpretation in adolescent patients.

**Fig. 1:**
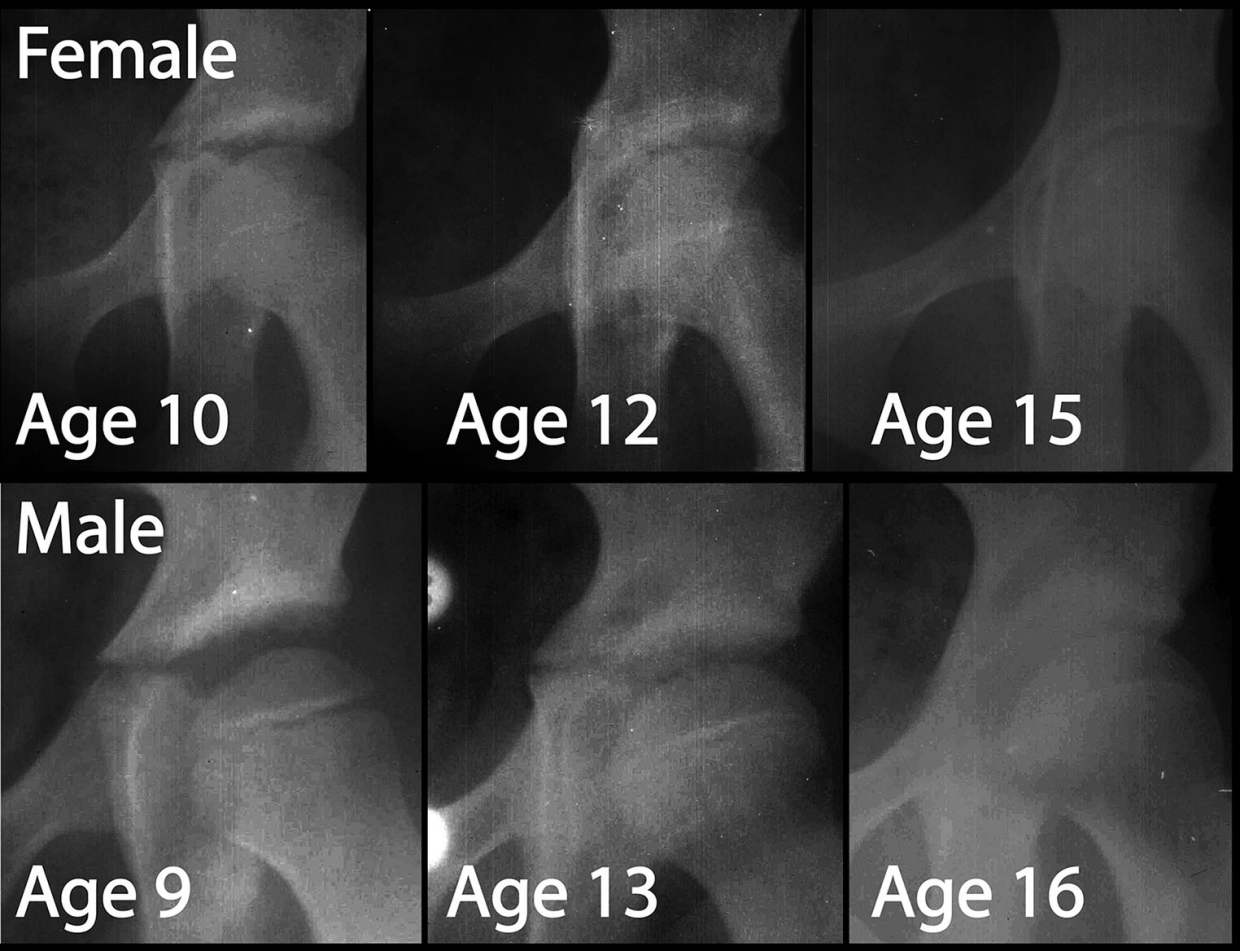
Appearance and remodeling of the ‘triradiate bump’ sign in an adolescent female (*top row*) and male (*bottom row*). This is a transient medial projection of the triradiate cartilage which mimics the ischial spine sign. The *top row* demonstrates how the bump resolves over a 5-year course, while the *bottom row* demonstrates how the bump becomes more prominent near the time of triradiate closure before resolving

We identified a novel radiographic sign (seen on AP plain films of the pelvis) where the triradiate cartilage transiently projects medially within the pelvic brim around the time of triradiate closure, mimicking the ISS (Fig. 1). The purpose of this study is to characterize this newly identified radiographic sign using a longitudinal radiographic study.

## Methods

**Fig. 2:**
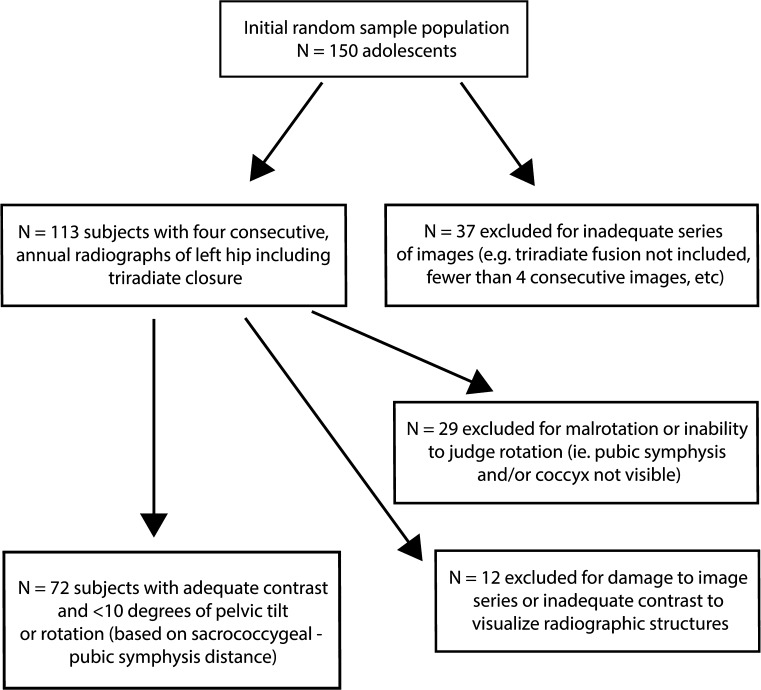
Flow chart characterizing the screening and inclusion/exclusion criteria for the study’s sample population

For the purpose of this study, we utilized the Brush Inquiry, a historical longitudinal radiographic study of healthy adolescents. The Brush Inquiry includes images of >4,000 male and female adolescents from the Greater Cleveland area enrolled between 1926 and 1942. All subjects in this study were enrolled either through referral by primary care physicians or through ‘health contests’ at local schools. These checkpoints served as a means to ensure that all subjects were free of obvious disease or deformity [12]. As part of an annual radiographic series, plain films of the left hip were obtained for each subject annually throughout their participation in the study. From the Brush Inquiry population, radiographic films from an initial random sample population of 150 subjects were uploaded to a digital database. Images from each subject were reviewed and subjects were excluded if their film series did not include at least four consecutive, annual AP radiographs of the left hip, including physeal closure. Subjects were also excluded if any of these radiographs were damaged, contained inadequate contrast or clarity, or did not contain the pubic symphysis in the field of view to allow for measurement of rotation. Pelvic rotation and tilt have been shown to affect coxometric indices [13–15], and specifically have been shown to influence radiographic interpretation of markers of acetabular retroversion [14–16]. A previous study by Kakaty et al. [16] confirmed that sensitivity and specificity of the ISS are maintained with pelvic rotation of up to 9° and pelvic tilt of up to 12°. Since the goal of this study was to characterize a radiographic sign which may mimic the ISS, we limited our evaluation of films to those in which one might expect to see a valid ischial spine sign, i.e., those with <10° of pelvic tilt or rotation. In order to accomplish this, we utilized standardized radiographic calibration markers present on each film and measurement of the sacrococcygeal joint to pubic symphysis (SCJ-PS) distance. Previous studies reveal that 2 cm of horizontal and vertical SCJ-PS distance correspond to 10° of pelvic rotation and tilt, respectively [14, 15]. All films were subsequently screened to confirm that they met these strict inclusion criteria. Figure 2 depicts a flow chart that characterizes the screening and inclusion criteria for the study’s sample population.

**Fig. 3:**
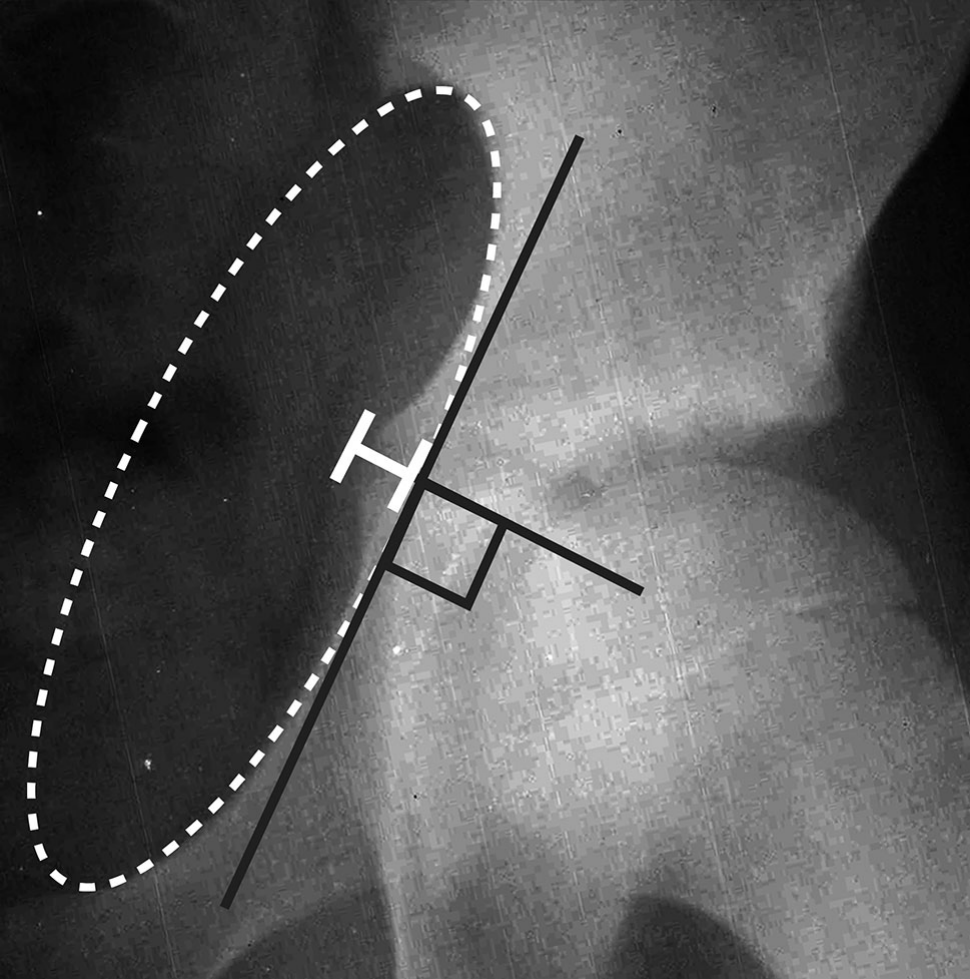
The prominence of the ‘triradiate bump’ is measured as the amount of medial projection of the triradiate cartilage (*white brackets*) from a point perpendicular to the tangent of a best-fit *ellipse* overlaying the iliopectineal line

**Fig. 4:**
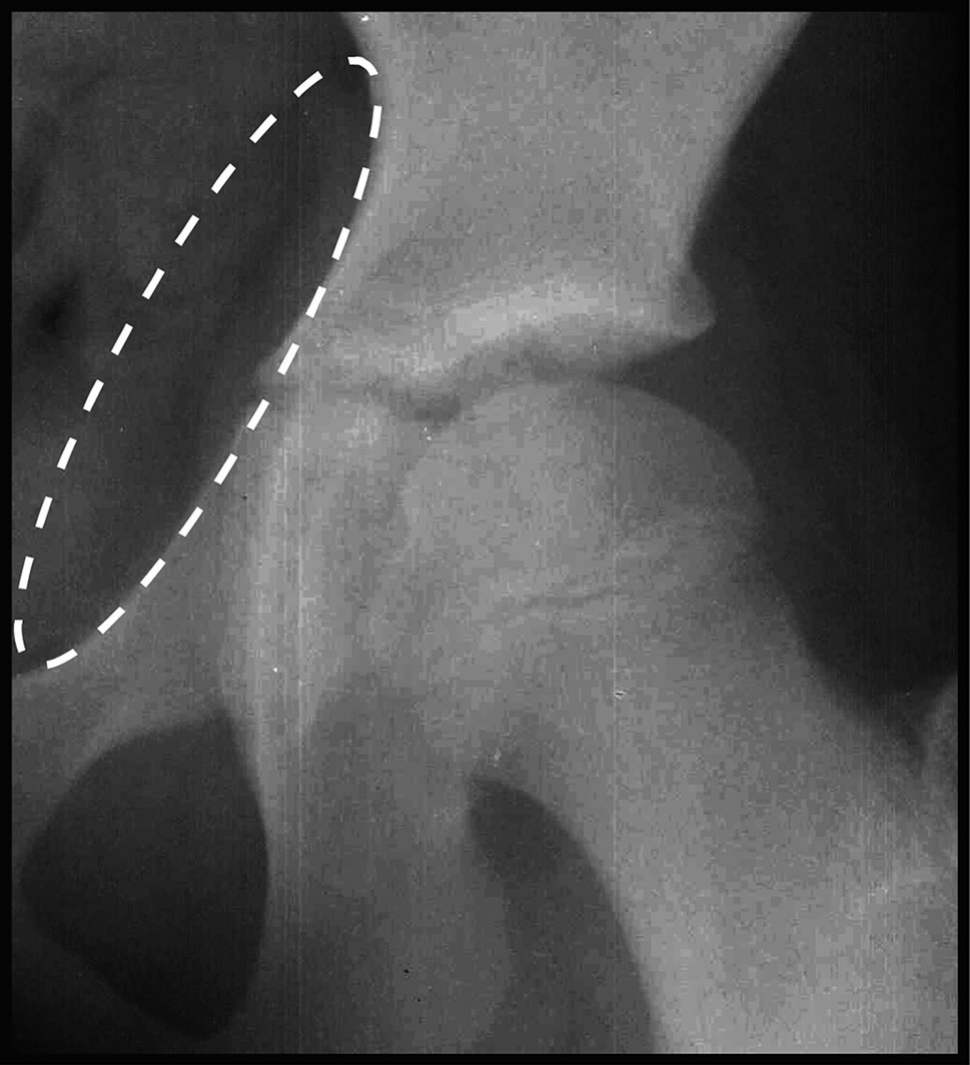
The minimum amount of medial projection of the triradiate cartilage considered a ‘positive’ triradiate bump sign was 3 mm. This cut-off was set as we noted medial lipping of the triradiate cartilage commonly broke the projected best-fit ellipse by 1–2 mm but did not reflect significant medial projection of the triradiate cartilage

From this initial sample population, we identified 72 subjects who met the above criteria. Images were reviewed to identify the presence of the ‘triradiate bump’, a transient medial projection of the triradiate cartilage within the pelvic brim around the time of physeal closure. We evaluated each series of films to determine the year at which the triradiate bump was most prominent, and the number of years relative to triradiate closure after which it had completely remodeled. All images were processed and measured with Image J software (http://imagej.nih.gov/ij/; National Institutes of Health, Bethesda, MD, USA). Triradiate bump prominence was measured by first overlaying a best-fit ellipse on the radiographic projection of the iliopectineal line. The prominence of the ‘triradiate bump’ was measured as the amount of medial projection of the triradiate cartilage (in mm) from a point perpendicular to the tangent of a best-fit ellipse overlaying the iliopectineal line. (Fig 3). The minimum amount of medial projection considered a positive ‘triradiate bump’ was 3 mm as we noticed that triradiate cartilage medial ‘lipping’ would commonly break the best-fit ellipse by 1–2 mm without reflecting significant medial projection of the triradiate cartilage (Fig. 4). Differences between genders were compared for rate of incidence, age at prominence, and remodeling relative to triradiate closure.

## Statistics

All statistical analyses were performed with SPSS (IBM Corp. Released 2015. IBM SPSS Statistics for Windows, Version 23.0. Armonk, NY: IBM Corp). Descriptive statistics (mean and standard deviation) were determined for each of the above variables. Comparison of the prevalence of the triradiate bump between genders was performed using the chi-squared test. Differences between genders in age at triradiate bump prominence, amount of medial projection (in mm), prominence relative to triradiate closure, and age at complete remodeling were compared using Student’s *t* tests. Significance for all tests was set at *p* < 0.05. Twenty plain films were re-measured by the initial author (WZM) and a second grader (RTL) to confirm the reliability of measurement of the medial projection of the triradiate bump. Inter- and intra-observer reliability was determined through determination of intraclass correlation coefficients (ICC). The results of the intraclass correlation coefficients were interpreted as follows—<0.40 was considered poor, 0.40–0.59 was considered fair, 0.60–0.74 was considered good, and >0.74 was considered excellent [17–19].

## Results

**Fig. 5:**
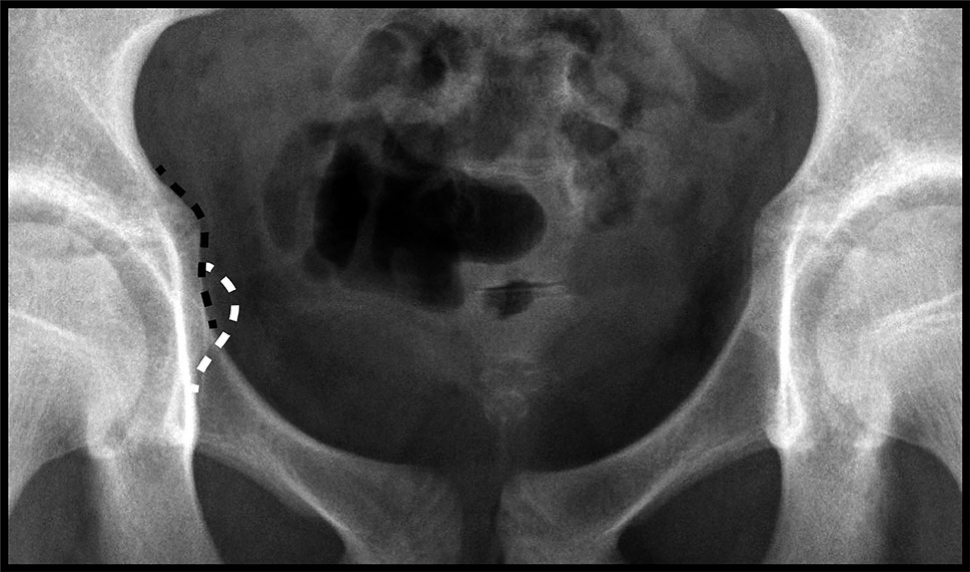
Anterior−posterior plain film of the pelvis of a female aged 11 years and 11 months demonstrating medial prominence of both the ischial spines (*white dashed line*) and the triradiate cartilages (‘triradiate bump’, *black dashed line*). Note the triradiate cartilage is located superior to the ischial spine and has a horizontal limb of radiolucency

A total of 423 AP plain films of the left hip were reviewed in 72 subjects (mean 5.9 ± 1.2 films per subject). There were 32 males and 40 females included in the study whose ages at the time of participation ranged from 10–18 years. The transient medial projection of the triradiate cartilage (‘triradiate bump’) was identified in 26/40 (65 %) females and 22/32 (69 %) males (*p* = 0.74). In all cases, the projection was qualitatively noted to be just superior to the ischial spine (Fig. 5). There was a wide range in the amount of medial projection of the triradiate bump from 3.2 to 8.1 mm; however, there was no significant difference in the amount of medial projection between males (5.1 ± 1.4 mm) and females (4.7 ± 0.8 mm, *p* = 0.29). The medial projection of the triradiate cartilage was most prominent at 10.8 ± 0.8 years of age in females and 12.6 ± 0.7 years of age in males (*p* < 0.001). This projection was most prominent at 2.1 ± 0.6 or 1.8 ± 0.7 years before triradiate closure for females and males, respectively (*p* = 0.15). The medial prominence of the triradiate cartilage completely remodeled in all cases at a mean of 0.1 ± 0.3 or 0.3 ± 0.5 years after triradiate closure in females and males, respectively (*p* = 0.22).

### Inter- and intra-oberserver reliability

Determination of intraclass correlation coefficients revealed excellent inter-observer agreement between the two authors (0.92) and excellent intra-observer agreement within the same grader’s measurement (0.95).

## Discussion

Radiographic assessment of acetabular retroversion remains central in the evaluation for FAI. Two radiographic signs have been identified on the AP pelvis film that signal acetabular retroversion—the COS and the ISS. The ISS is thought to be present in between 7 and 30 % of asymptomatic subjects [20, 21] and was proposed as a simpler alternative to the COS where clinicians only needed to identify “a triangular-shaped radio-opaque structure that points medially from the pelvic brim toward the pelvic inlet” [11]. Although the ISS represents a useful radiographic tool in the assessment of acetabular version, we have demonstrated that it should be interpreted cautiously in the adolescent hip.

The transient medial projection of the triradiate cartilage within the pelvic brim (the ‘triradiate bump’ sign) is a common radiographic finding in healthy adolescents which may mimic prominence of the ischial spine. Through the use of a longitudinal radiographic study, we demonstrated that the triradiate cartilage projects medially within the pelvic brim in the majority of healthy adolescents. This medial prominence peaks approximately 2 years before triradiate closure and then completely remodels around the time of triradiate closure. This finding was similarly present in both males and females.

Misinterpretation of the medial prominence of the triradiate cartilage as an ISS may lead to the false assumption that a patient has acetabular retroversion. However, the two signs can be distinguished as the projection of the ischial spine is located more inferiorly within the pelvic brim. In addition, the triradiate cartilage can be visualized within the triradiate bump, and thus a triradiate bump has a horizontal limb of radiolucency extending to its medial border (Fig. 5). Finally, the presence or absence of the COS, another indication of acetabular retroversion, could be used to further evaluate version. Recognition of these radiographic markers will help distinguish the ISS from the triradiate bump and prevent misinterpretation of acetabular version in the adolescent hip.

Pelvic rotation or tilt can also lead to misinterpretation of films and radiographic signs, including the ISS and the COS [16, 22]. Consequently, one of the most important tools in radiographic evaluation of the adolescent hip is recognition of a proper AP pelvis film. A proper film is obtained with the patient supine with their legs internally rotated 15° and the X-ray tube placed 120 cm away from the film, centered on the midpoint between the pubic symphysis and a line connecting the two anterior superior iliac spines [23]. Although there are slight variations between genders, an appropriate AP pelvis film should position the tip of the coccyx approximately 1–3 cm from the pubic symphysis. This technique was used in our study to confirm appropriate pelvic tilt and rotation. Additionally, symmetry of the obturator for aminae, iliac wings, and radiographic teardrops are also useful markers of appropriate positioning [22]. Using these radiographic cues, one can avoid the pitfalls of misinterpretation of pelvic radiographs.

The strengths and limitations of this study both stem from the use of a historical longitudinal radiographic collection. The use of the Brush Inquiry afforded us the unique opportunity to study the nuanced radiographic development of the adolescent hip through annual plain films of the left hip from childhood through to skeletal maturity. Characterization of the presence and remodeling of the triradiate bump would not have otherwise been possible. However, the use of these historical films was at times limited by poor contrast or rotation of the films. Our strict inclusion criteria of adequate contrast and <10° of pelvic rotation or tilt (as judged by the sacrococcygeal-pubic symphysis distance) helped to assure that only quality AP films of the left hip were included the study. Furthermore, since the study utilized AP films of the hip rather than AP pelvis films, differences in beam centering may lead to slight variations in radiographic anatomy that may have influenced our measurements; however, we have identified the same radiographic findings on AP pelvis films (Fig. 5) and suspect these influences are minimal. Finally, although these films were obtained approximately 80 years ago (1926–1942), we believe the data are still relevant as the development of a adolescent hip appears to follow a similar timeline in current radiographic studies [24] compared to studies using this collection [25, 26].

It is not entirely clear why the triradiate edge becomes so prominent two years before triradiate closure. We are not aware of any other growth plates within the body which behave similarly. However, other morphological changes such as physeal cupping have been described in the years preceding maturity of the physis. In the proximal and distal femur, cupping has been proposed as a physiologic mechanism to stabilize the physis during a time when the undulations within the physis are decreasing in relative size with growth [27–29]. It is possible that the extension of the triradiate cartilage into the medial pelvis represents a similar mechanism to confer stability to the growth plate.

In summary, we demonstrated that the transient medial prominence of the triradiate cartilage (the ‘triradiate bump’ sign) occurs regularly in healthy adolescent hips just before triradiate closure. This medial projection within the pelvic brim may mimic the ISS and falsely suggest the presence of acetabular retroversion. However, the two radiographic signs can be distinguished by the fact that the triradiate bump is located superior to the ischial spine, has a horizontal limb of radiolucency (from the triradiate cartilage), and completely remodels around the time of triradiate closure. Although we continue to recommend the use of plain films of the pelvis as an initial radiographic study in the adolescent evaluation for FAI, we recommend close scrutiny of radiographs in this unique population to prevent misinterpretation of radiographic markers of acetabular retroversion.
